# Selection of valid reference genes for quantitative real-time PCR in *Cotesia chilonis* (Hymenoptera: Braconidae) exposed to different temperatures

**DOI:** 10.1371/journal.pone.0226139

**Published:** 2019-12-26

**Authors:** Qiu-Yu Li, Zi-Lan Li, Ming-Xing Lu, Shuang-Shuang Cao, Yu-Zhou Du

**Affiliations:** 1 School of Horticulture and Plant Protection & Institute of Applied Entomology, Yangzhou University, Yangzhou, China; 2 Joint International Research Laboratory of Agriculture and Agri-Product Safety, the Ministry, Yangzhou, China; IRIG-CEA Grenoble, FRANCE

## Abstract

In quantitative real-time PCR (qRT-PCR), data are normalized using reference genes, which helps to control for internal differences and reduce error among samples. In this study, the expression profiles of eight candidate housekeeping genes, 18S ribosomal (*18S rRNA*), elongation factor (*EF1*), glyceraldehyde-3-phosphate dehydrogenase (*GAPDH*), ribosomal protein L10 (*RPL10*), ribosomal protein L17 (*RPL17*), histone 3 (*H3*), arginine kinase (*AK*), amd β-Actin (*ACTB*), were evaluated in the parasitic wasp *Cotesia chilonis* in response to different temperatures. Specifically, the performance and stabilities of these genes were compared in adult wasps maintained in a growth condition at 27°C (normal storage conditions) and in adults obtained from pupae refrigerated at 4°C for five days (cold storage conditions). Data were analyzed using the ΔCt method, BestKeeper, NormFinder, and geNorm. The optimal numbers and stabilities of reference genes varied between the two temperature treatments (27°C and 4°C). In samples stored at normal developmental temperature (27°C), the requirement for normalization in response to low temperature exposures was three genes (*18S*, *H3*, *AK*), whereas normalization in response to high temperature exposures required only two reference genes (*GAPDH*, *ACTB*). In samples stored at cold temperature (4°C), for low temperature exposures two reference genes (*RPL17*, *RPL10*) were required for standardization, while following high temperature exposures three reference genes (*18S*, *H3*, *ACTB*) were needed. This study strengthens understanding of the selection of reference genes before qRT-PCR analysis in *C*. *chilonis*. The reference genes identified here will facilitate further investigations of the biological characteristics of this important parasitoid.

## Introduction

*Cotesia chilonis* (Matsumura) (Hymenoptera: Braconidae) is native to parts of southeastern and eastern Asia [1, 2]. *Cotesia chilonis* is the dominant parasitoid of the rice stem borer, *Chilo suppressalis* (Walker) (Lepidoptera: Pyralidae) larvae, which is a serious pest in China, particularly in the Yangtze River area and southern regions of China. *C*. *chilonis* is also an important biological control agent of some other stem borers and was previously imported into several African countries as a biological control agent [3, 4, 5]. Related biological characteristics of *C*. *chilonis* have been studied in previous researches, such as fecundity, parasitism rate, and sex ratio [6, 7]. Besides, there are few studies about the storage methods of C. chilonis, so we want to explore low temperatures to extend its shelf life so that it can be better utilized in the future.

Quantitative real-time PCR (qRT-PCR) was initially described in 1992 [8] and has become a widely used approach to analyze gene expression due to it’s accuracy, high reproducibility and sensitivity [9, 10, 11, 12, 13]. Unfortunately, variation in RNA isolation, cDNA quantification, transcription, and amplification can cause qRT-PCR to be error-prone [14, 15, 16, 17]. To correct this problem, reference genes are widely used to normalize qRT-PCR data, which can reduce error among samples and control for internal differences [18, 19]. However, no one gene exhibits constant expression under all test conditions; therefore, computational programs such as BestKeeper [16], NormFinder [20], and geNorm [21] are used to evaluate the performance of reference genes and identify the optimal set of reference genes for normalization for a particular species or biological sample [22, 23].

A number of reference genes are commonly used in the literature, especially housekeeping genes [14, 22, 23, 24, 25, 26, 27, 28, 29, 30, 31] such as *18S* (18S rRNA), *TUB* (encoding tubulin), and *ACTB* (*β*-actin). Many of these genes are involved in cellular maintenance and structural functions, and it is often assumed that they are expressed uniformly regardless of test conditions. However, the expression of these reference genes can, in fact, vary depending on test conditions [32, 33]. In this study, we evaluated eight candidate reference genes (*18S rRNA*, *ACTB*, *GAPDH*, *EF1*, *H3*, *RPL17*, *RPL10*, and *AK*) for expression stability in *C*. *chilonis* samples stored either at 27 ^o^C or 4 ^o^C and subjected to different temperatures. The reference genes identified in this study provide useful information on the ecological adaptation of this parasitoid wasp and will promote future research of gene expression.

## Materials and methods

### Biological samples

*Chilo suppressalis* and *C*. *chilonis* were collected from a suburb of Yangzhou (32.39°N, 119.42°E) and maintained in a laboratory growth chamber at 27 ± 1°C with a 16:8 h (light/dark) photoperiod and 60–70% RH [7]. *Chilo suppressalis* larvae were reared on six- to seven-day-old rice plants. The parasitoid wasps, *C*. *chilonis*, were reared on *C*. *suppressalis* larvae. Adults of both insect species were supplied with 10% honey solution. No specific permission was required for these activities, and the field studies did not involve endangered or protected species.

### Temperature treatments

Samples Stored at Normal Temperatures (27°C): cocoons and adults of *C*. *chilonis* were reared in a growth chamber maintained at a constant temperature of at 27 ± 1°C. Adults were then exposed to -13, -12, -9, -6, -3, 0, 3, 6, 9, 12, 15, 18, 21, 24, 27, 30, 33, or 36°C for 1 h prior to RNA extraction. Each experimental group contained 20 adults, and all experiments were repeated three times.

Samples Stored at Cold Temperatures (4 ^o^C): cocoons of *C*. *chilonis* were stored at 4 ^o^C for five days and then transferred to an environmental growth maintained at 27 ± 1°C. Adults were collected after eclosion, and then exposed to the above temperature. Each experimental group contained 20 adults, and all experiments were repeated three times.

### Cloning of selected reference genes

The eight housekeeping genes (*18S rRNA*, *EF1*, *GAPDH*, *RPL10*, *RPL17*, *H3*, *AK*, and *ACTB*) were amplified from *C*. *chilonis*, cloned, and sequenced (Table 1). Primer Premier 5 software was used to design primer pairs for subsequent amplification of genes from *C*. *chilonis* (http://www.premierbiosoft.com/primerdesign/index.html). Sequences for the eight primers, calculation efficiency (E), and Tm values are shown in Table 1.

**Table 1:** Primer sequences and characteristics of amplified reference genes.

Gene	Primer pair^*a*^	Primer sequences (5’-3’)	Amplicon (bp)	E^*b*^ (%)	Tm (°C)	R^2^^*c*^
*18S rRNA*	*18S rRNA*qRT-F *18S rRNA*qRT-R	AACTGGGGGCATTCGTATTG CTTTCGCTGATGTTCGTCTTG	72	91	54	0.998
*EF1*	*EF1*qRT-F *EF1*qRT-R	TTGCCTTCGTCCCCATCTCT ACGTTCAACCTTCCATCCCT	92	97.8	55.5	0.994
*GAPDH*	*GAPDH*qRT-F *GAPDH*qRT-R	GAAGGTGGTGCCAAGAAAG GCATGGACAGTGGTCATAAGA	203	106.7	58.1	0.978
*H3*	*H3*qRT-F *H3*qRT-R	CGTCGCTCTTCGTGAAATCA TCTGGAAACGCAAGTCGGTC	122	97.4	58.1	0.978
*RPL10*	*RPL10*qRT-F *RPL10*qRT-R	CCCAAAATCCCGTTTCTGT CTGGCTTTCTTCTTTCCCAA	121	99.9	56.7	0.976
*RPL17*	*RPL17*qRT-F *RPL17*qRT-R	AGGCTGTAATGGAACACAAAGA GCAAAAGCTGGAGCAAAAAT	141	91.6	56.1	0.983
*ACTB*	*ACTB*qRT-F *ACTB*qRT-R	AAAAGCCAACCGTGAGAAGAT CAGTGGTACGACCAGAAGCG	113	105.6	56.5	0.990
*AK*	*AK*qRT-F *AK*qRT-R	GTTCGGTTTCTTGACCTTCTG TCGAGCTTGGCTTTGTTG	102	100.6	56.7	0.980

^*a*^ F and R refer to forward and reverse primers, respectively.

^*b*^ Real-time qPCR efficiency (calculated from the standard curve).

^c^ Coefficient of determination.

### Quantitative real-time PCR analysis

Total RNA was extracted from *C*. *chilonis* with the SV Total RNA isolation system (Promega, USA), followed by DNase treatment to eliminate DNA contamination. RNA integrity of the samples was confirmed by comparing the ribosomal RNA bands in ethidium bromide-stained gels. RNA purity was measured by spectrophotometry at *A*_260_ and *A*_280_ (Eppendorf Biophotometer). To confirm consistent amounts of cDNA, RNA concentrations were measured twice for each sample. Reverse transcription of RNA (0.5 μg) into first strand cDNA was carried out using the Bio-Rad iScriptTM cDNA Synthesis Kit (Bio-Rad, USA). Real-time PCR reactions were performed in a 20 μl reaction volume containing 10 μl Bio-Rad iTaqTM Universal SYBR® Greensupermix (2x) (Bio-Rad Laboratories, Berkeley, USA), 1 μl (10 μM) of each gene-specific primer (Table 1), 2 μl cDNA template and 6 μl of water. Reactions were performed with a Bio-Rad CFX-96 real-time PCR system as follows: 3 min of polymerase activation at 95 ^o^C, followed by 40 cycles of denaturation at 95 ^o^C for 30 s, and annealing for 30 s at the Tm for each gene (Table 1). Melting curve analysis from 65 ^o^C to 95 ^o^C was conducted to evaluate the specificity of the amplified PCR products. Each treatment included three replicates, and each reaction was performed in triplicate.

### Data analysis

Data were analyzed with Bio-Rad CFX Manager TM 3.1 software. The threshold cycle (Ct value) represents the first cycle where the fluorescence signal is significantly different in comparison with the background. Average Ct values were calculated based on all biological replicates. Expression stability of the eight candidate reference genes was evaluated using the geNorm (http://medgen.ugent.be/jvdesomp/genorm/index.php) [34], ΔCt method [35], NormFinder (http://www.mdl.dk/publicationsnormfinder.htm) [20], and and BestKeeper (http://www.wzw.tum.de/genequantification/bestkeeper.html) [16]. The ΔCt method utilizes relative pair-wise comparisons, and standard deviations (SD) are used to rank the stability. geNorm calculates the expression stability value (M) of each gene and then performs pair-wise comparison (Vn/Vn+1) of individual genes with other genes [34]. NormFinder uses a model-based approach to estimate expression variation in the selection of suitable reference genes [20]. Genes with the lowest values are the most stable. BestKeeper can select the optimal reference gene and sort the genetic stability by inputting the original Ct value and the PCR efficiency E value.

## Results

### Validation of PCR assays

All PCR assays resulted in production of a single amplicon, ranging in size from 72 to 203 bp, depending on the primer pair used (Table 1). Furthermore, a single, sharp peak was confirmed in melting curve analysis for each amplicon. A standard curve was generated for each gene, using eight, 10-fold serial dilutions (10^−0^ to 10^−7^) of the pooled cDNAs with Bio-Rad CFX Manager^TM^ 3.1 software. All genes displayed efficiency (E values) between 91–106.7% (Table 1).

### Expression profiles of candidate reference genes

**Fig 1:**
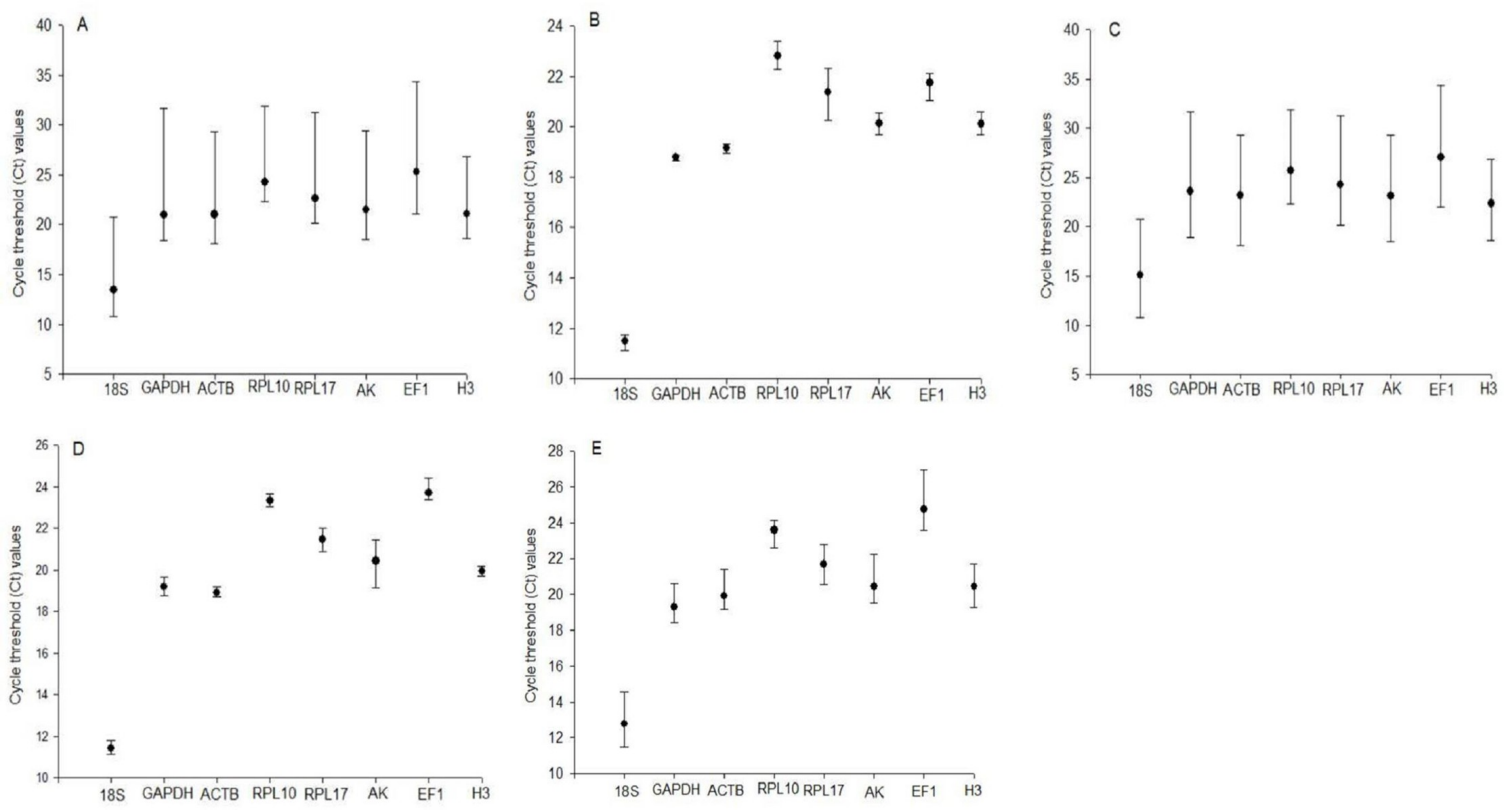
Expression stability of candidate reference genes in *C*. *chilonis* exposed to different temperatures. (A) All *C*. *chilonis* samples (*n* = 272). Samples stored at 27°C and then exposed to (B) high temperatures (*n* = 32) or (C) low temperatures (*n* = 104). Ct values of reference genes in *C*. *chilonis* subjected to cold storage (4°C) and then exposed to (D) high temperatures (*n* = 40) or (E) low temperatures (*n* = 112). Solid circles denote the mean of duplicate samples and the bars indicate minimum to maximum values.

The mean Ct values of the eight reference genes ranged from 13.25 (*18S*) to 25.13 (*EF1*). *H3* showed the smallest Ct value among all the experimental samples followed by *RPL10*, *18S*, *AK*, *RPL17*, *ACTB*, *GAPDH*, and *EF1* (Fig 1A). However, in samples stored at 27 ^o^C (normal conditions) and exposed to high and low temperatures, the smallest Ct values were observed for *GAPDH* (Fig 1B) and *H3* (Fig 1C), respectively. In samples stored at 4 ^o^C and exposed to high and low temperatures, *H3* (Fig 1D) and *RPL10* (Fig 1E) showed the smallest Ct variations, respectively. In summary, no specific reference gene is suitable for all experimental treatments.

### Gene expression in samples stored at 27 ^o^C

geNorm was used to calculate the mean expression stability (*M* values) and to plot the effects of different factors by using pairwise comparisons. The most unstable gene showed the highest M value and was subsequently excluded. In samples stored at 27 ^o^C (normal conditions) and then exposed to low temperatures, the ΔCt method showed that the most reference genes with the most stable rates of expression were *18S/ACTB* (SD = 1.329) and the least stable reference gene was *EF1* (SD = 1.735) (Table 2). However, according to the results of geNorm, the stability ranking from the most to the least stable was *18S+H3* > *AK* > *GAPDH* > *ACTB* > *RPL10* > *EF1* > *RPL17*, which was consistent with results obtained with Normfinder. BestKeeper identified *H3* as the reference gene with the most stable expression stability at low temperatures (Table 3).

**Table 2:** Ranking of candidate reference genes of *Cotesia chilonis* stored at 27 ^o^C and then subjected to low temperatures.

Conditions	Rank	ΔCt		BestKeeper		Normfinder		geNorm	
		Gene name	Standard deviation	Gene name	Standard deviation	Gene name	Stability value	Gene name	Stability value
**Low temperature**	1	ACTB/18S	1.329	H3	3.083	18S	0.649	18S/H3	1.164
	2			RPL10	3.374	H3	0.732		
	3	RPL10	1.436	AK	3.674	ACTB	0.815	AK	1.531
	4	AK	1.445	RPL17	3.712	RPL10	0.986	GAPDH	1.749
	5	H3	1.461	18S	3.925	GAPDH	0.994	ACTB	1.819
	6	GAPDH	1.590	ACTB	4.324	AK	1.062	RPL10	1.866
	7	RPL17	1.702	GAPDH	4.583	EF1	1.462	EF1	1.971
	8	EF1	1.735	EF1	4.705	RPL17	1.638	RPL17	2.145

Expression stability was measured using the **Δ**Ct method, BestKeeper, Normfinder, and geNorm and then ranked from most to least stable.

**Table 3:** Ranking of candidate reference genes of *Cotesia chilonis* stored at 27 ^o^C and then exposed to high temperatures.

Conditions	Rank	ΔCt		BestKeeper		Normfinder		geNorm	
		Gene name	Standard deviation	Gene name	Standard deviation	Gene name	Stability value	Gene name	Stability value
**High temperature**	1	GAPDH	0.330	GAPDH	0.063	GAPDH	0.138	GAPDH/ACTB	0.254
	2	H3	0.360	ACTB	0.140	RPL10	0.168		
	3	ACTB	0.362	AK	0.231	18S	0.178	18S	0.306
	4	RPL10	0.366	18S	0.253	ACTB	0.211	RPL10	0.363
	5	18S	0.378	RPL10	0.307	H3	0.255	H3	0.395
	6	AK	0.438	H3	0.314	AK	0.329	AK	0.431
	7	EF1	0.446	EF1	0.340	EF1	0.338	EF1	0.457
	8	RPL17	0.637	RPL17	0.654	RPL17	0.548	RPL17	0.552

Expression stability was measured using the **Δ**Ct method, BestKeeper, Normfinder, and geNorm and then ranked from most to least stable.

In samples stored at 27 ^o^C and then exposed to high temperatures, the ΔCt method indicated that *GAPDH* (SD = 0.330) was the most stable, and *RPL17* (SD = 0.637) was the least stable reference genes (Table 3). geNorm analysis ranked reference gene stability as *GAPDH+ACTB* >*18S* > *RPL10* >*H3* > *AK* > *EF1* > *RPL17*, which was similar to results from Normfinder and BestKeeper.

In conclusion, when samples were stored at 27 ^o^C and then exposed to high temperatures, the most stable reference gene was *GAPDH* (Table 3). However, when samples were exposed to low temperatures, *18S* showed greater expression stability (Table 2).

### Gene expression in samples stored at 4 ^o^C

For samples stored at 4 ^o^C and then exposed to low temperatures, geNorm ranked reference gene expression stability as *RPL10/RPL17* > *H3* > *GAPDH* > *ACTB* > *AK* > *18S* > *EF1*. However, the ranking obtained with BestKeeper was slightly different. According to the ΔCt method, *GAPDH* (SD = 0.494) was the most stable and *EF1* (SD = 0.682) was the least stable reference gene, respectively (Table 4). In samples stored at 4 ^o^C and then exposed to high temperatures, the ΔCt method, geNorm, BestKeeper, and Normfinder all identified *H3* as the most stable and *AK* as least stable reference gene, respectively (Table 5). geNorm ranked the overall expression stability order as *18S+H3* > *ACTB* > *RPL10* > *RPL17* > *EF1* > *GAPDH* > *AK*. In conclusion, under low temperature conditions the most stable reference gene was *GAPDH* (Table 4), while under high temperature conditions *H3* showed more stability (Table 5).

**Table 4:** Ranking of candidate reference genes of *Cotesia chilonis* stored at 4 ^o^C and then subjected to low temperatures.

Conditions	Rank	ΔCt		BestKeeper		Normfinder		geNorm	
		Gene name	Standard deviation	Gene name	Standard deviation	Gene name	Stability value	Gene name	Stability value
**Low temperature**	1	GAPDH	0.494	RPL10	0.370	H3	0.255	RPL10/RPL17	0.451
	2	H3	0.501	GAPDH	0.382	GAPDH	0.257		
	3	ACTB	0.521	RPL17	0.530	ACTB	0.264	H3	0.504
	4	AK	0.564	ACTB	0.565	18S	0.378	GAPDH	0.572
	5	18S	0.585	H3	0.604	AK	0.388	ACTB	0.630
	6	RPL10	0.599	AK	0.663	RPL10	0.411	AK	0.656
	7	RPL17	0.616	18S	0.670	RPL17	0.412	18S	0.687
	8	EF1	0.682	EF1	0.758	EF1	0.537	EF1	0.741

Expression stability was measured using the **Δ**Ct method, BestKeeper, Normfinder, and geNorm and then ranked from most to least stable.

**Table 5:** Ranking of candidate reference genes of *Cotesia chilonis* stored at 4 ^o^C and then subjected to high temperatures.

Conditions	Rank	Δ Ct		BestKeeper		Normfinder		geNorm	
		Gene name	Standard deviation	Gene name	Standard deviation	Gene name	Stability value	Gene name	Stability value
**High temperature**	1	H3	0.299	H3	0.097	H3	0.089	H3/18S	0.201
	2	18S	0.312	ACTB	0.125	18S	0.095		
	3	ACTB	0.337	18S	0.179	ACTB	0.208	ACTB	0.263
	4	RPL10	0.361	RPL10	0.240	EF1	0.219	RPL10	0.299
	5	EF1	0.363	RPL17	0.273	RPL17/GAPDH	0.221	RPL17	0.324
	6	GAPDH	0.376	EF1	0.290			EF1	0.356
	7	RPL17	0.393	GAPDH	0.322	RPL10	0.243	GAPDH	0.381
	8	AK	0.698	AK	0.727	AK	0.569	AK	0.502

Expression stability was measured using the **Δ**Ct method, BestKeeper, Normfinder, and geNorm and then ranked from most to least stable.

### Optimal numbers of reference genes for normalization

**Fig 2:**
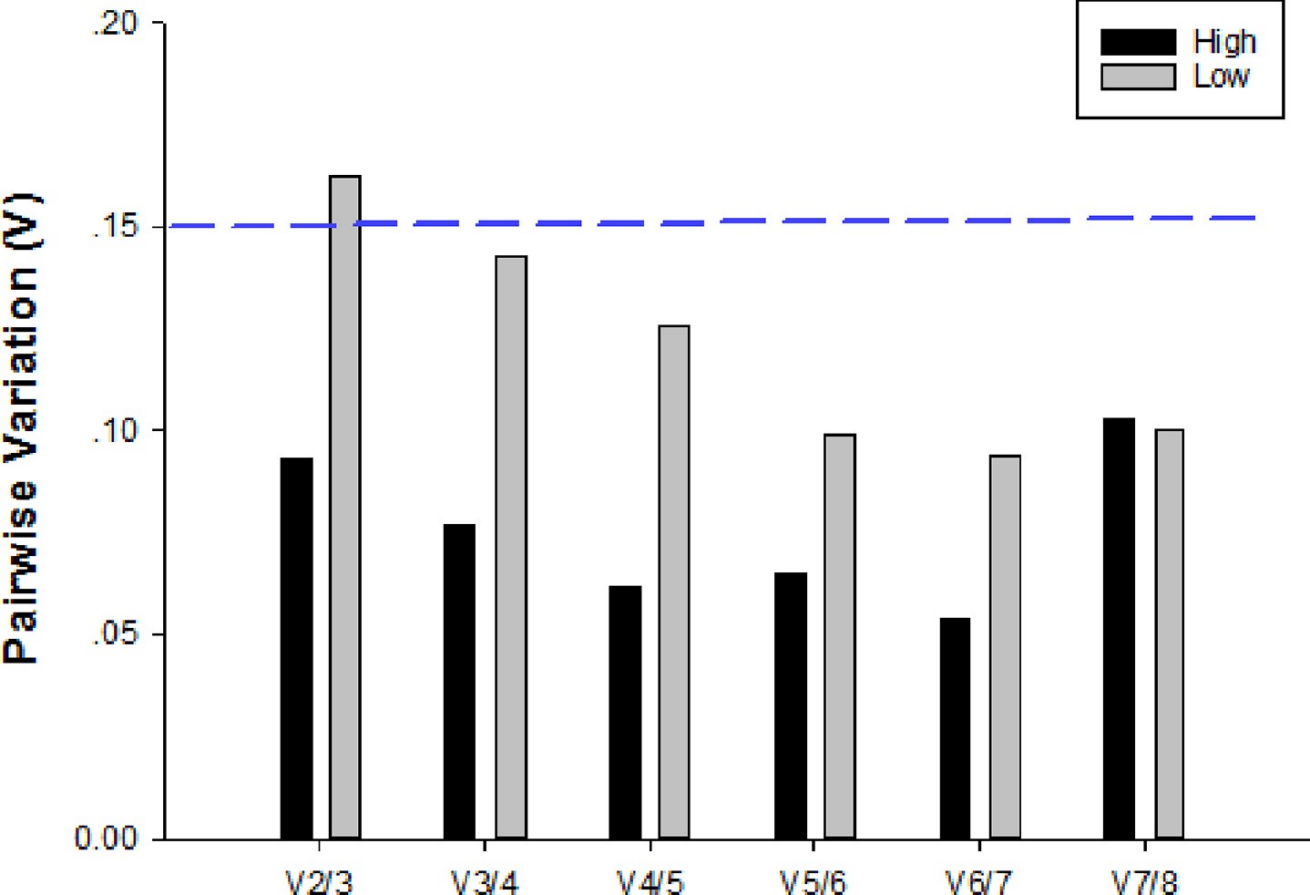
Optimal number of reference genes for normalization in *C*. *chilonis* stored at 27°C. Pairwise variation (Vn/Vn+1) was analyzed between normalization factors NFn and NFn+1 by geNorm to determine the optimal number of reference genes. When values fell below the 0.15 cut-off value (dashed line), additional genes were not required for normalization of gene expression.

Pairwise variation (Vn/Vn+1) was calculated by geNorm. In samples stored at 27 ^o^C, analysis of exposure to low temperatures showed that pairwise variation values for V3/4 were below the proposed cut-off value of 0.15; therefore, three reference genes were optimal (Fig 2). Interestingly, all V ratios from samples stored at 27 ^o^C and exposed to high temperatures were below the value of 0.15; thus, additional genes were not required. Therefore, in samples at 27 ^o^C, the required number of reference genes for low and high temperatures was three (*18S*, *H3*, *AK*) and two (*GAPDH*, *ACTB*), respectively.

**Fig 3:**
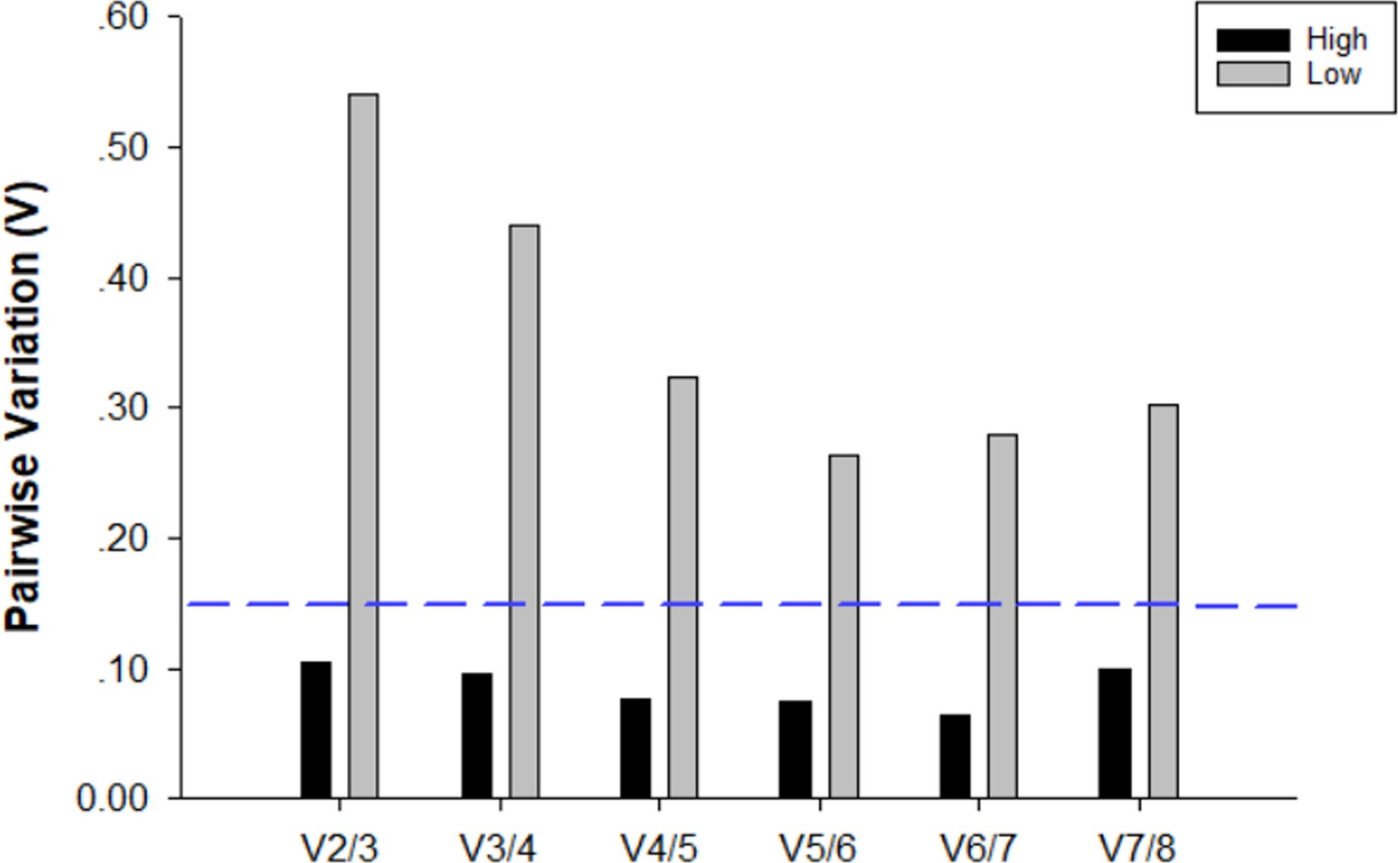
Optimal number of reference genes for normalization in *C*. *chilonis* stored at 4°C. Pairwise variation (Vn/Vn+1) was analyzed between normalization factors NFn and NFn+1 by *geNorm* to determine the optimal number of reference genes. When values fell below the 0.15 cut-off, additional genes were not required for normalization of gene expression.

In samples stored at 4 ^o^C and exposed to low temperatures, geNorm analysis indicated V ratios that were above the proposed cut-off value of 0.15 (Fig 3). In contrast, all the pair-wise variation values of samples exposed to high temperatures fell below the 0.15 cut-off, and normalization of these samples required two reference genes (Fig 3). According to the geNorm manual, the threshold of 0.15 must not be taken as an absolute cut-off, and three best reference genes is in most cases a valid normalization strategy. In summary, for samples stored at 4 ^o^C, the requirement for exposure to low and high temperatures was three (*RPL17*, *RPL10*, *H3*) and two (*18S*, *H3*) reference genes, respectively.

## Discussion

qRT-PCR is an accurate and sensitive technique to semi-quantitative PCR and northern blot hybridizations for gene expression [36,37]; however, in the design of effective qRT-PCR studies it is essential to select the appropriate reference gene [12, 13, 17, 20, 38, 39, 40], and multiple reference genes were used to improve the analysis of gene expression [17, 22, 23, 41]. This is the first study to define suitable reference genes for RT-qPCR in *C*. *chilonis*. Here, we used the ΔCt method and three Excel-based algorithms, e.g. geNorm, Normfinder and BestKeeper, to evaluate the stability of eight potential reference genes in *C*. *chilonis* stored at 27 or 4 ^o^C.

In *C*. *chilonis* stored at 27 ^o^C and exposed to low temperatures, the ideal reference genes were *18S*, *H3*, and *AK* (according to geNorm), *18S* (per Normfinder) and *H3*, *RPL10*, *AK* and *18S* (based on BestKeeper). When these results are considered together, *18S* emerges as a consensus reference gene for samples exposed to low temperatures. V2/3 values < 0.15 indicated that three stably-expressed genes were required for normalization of samples stored at 27 ^o^C. *GAPDH* emerged as the most stable reference gene based on the three algorithms when samples stored at 27 ^o^C were exposed to high temperatures.

When *C*. *chilonis* was stored at 4 ^o^C and exposed to low temperatures, *RPL17* and *RPL10* emerged as stable reference genes using geNorm, *H3* was identified based on Normfinder, and *RPL10* and *GAPDH* were identified by BestKeeper. Collectively, these results suggest that *H3* and *RPL17* are the most suitable reference genes for samples stored at 4 ^o^C.

In *Apis mellifera*, the best reference genes were *RPL49*, *EF1*, *AK* and *GAPDH* [42,43,44], and AK was found most stable in other insects, such as *Bombus lucorum*, *Spodoptera litura* [45, 46]. However, expression of these genes was too unstable to be used for normalization in *C*. *chilonis*. In our study, both the identity and number of reference genes were different for samples stored at 27 and 4 ^o^C. EF1 catalyzes GTP-dependent binding of aminoacyl-tRNA to ribosome receptor sites and has been widely used as a reference gene for insects [32, 41, 47]. However, *EF1* was not a suitable reference gene in the current study. *18S* has long been considered an ideal reference gene due to the fact that the expression level of rRNA varies less than mRNA [9, 48, 49]. Furthermore, in four coccinellid species, *18S* was stably expressed throughout the majority of biotic and abiotic conditions. However, in our study, *18S* was a useful reference gene for samples stored at 27 ^o^C but was less reliable in experiments conducted with samples stored at 4 ^o^C. This result is consistent with those of earlier studies where *18S* was used in different tissues of *Bactrocera dorsalis* and to study physiological responses in *Drosophila melanogaster* [50].

In summary, we evaluated the stability of eight candidate reference genes for use in qRT-PCR studies of *C*. *chilonis*. This study provides a standardized procedure for quantification of gene expression in *C*. *chilonis* and also identifies parameters that must be considered when studying gene expression in this species. Our results will facilitate future qRT-PCR experiments of this parasitoid and will provide valuable data on the most suitable genes to use when the insect is stored at 27 or 4 ^o^C.
